# Supplementary material for: Immune response and endocytosis pathways are associated with the resilience against Alzheimer’s disease
Source: Transl Psychiatry. 2020 Sep 29;10:332. doi: 10.1038/s41398-020-01018-7 (PMC7524800; doi:10.1038/s41398-020-01018-7)
Supplement: Supplementary file 2 — Supplementary Figures [file 41398_2020_1018_MOESM2_ESM.pdf]

Supplementary Figures

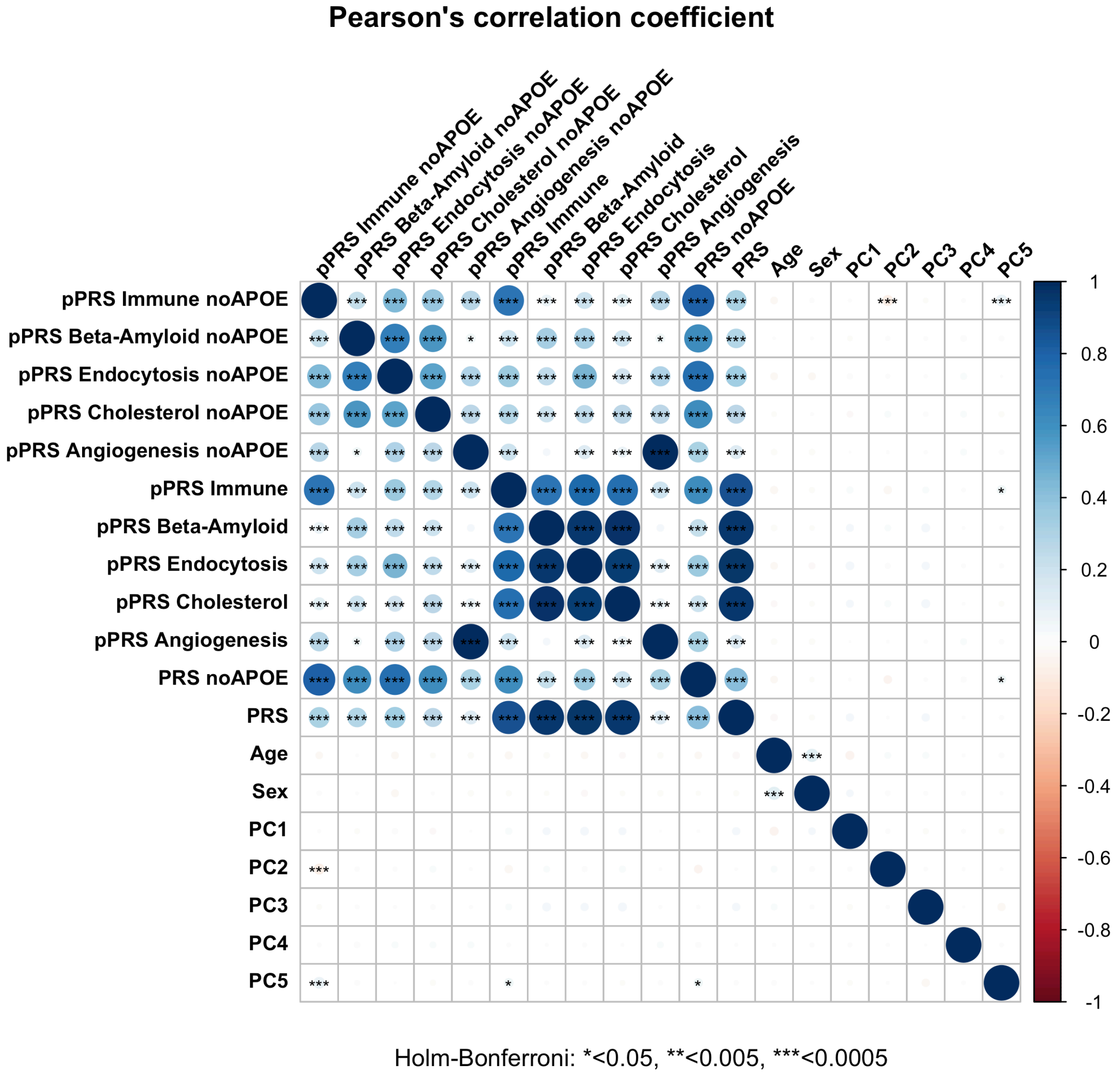

Figure S1: Correlation plot of the (p)PRS and covariates: the figure shows the correlation between the pathway-PRS (respectively with and without *APOE* variants), the full-PRS (respectively with and without *APOE* variants), ages (ages at study inclusion for controls, ages at diagnosis for AD cases) and the 5 principal components derived from the population stratification analysis. We used Pearson correlation and *p-values* were adjusted with Holm-Bonferroni correction.

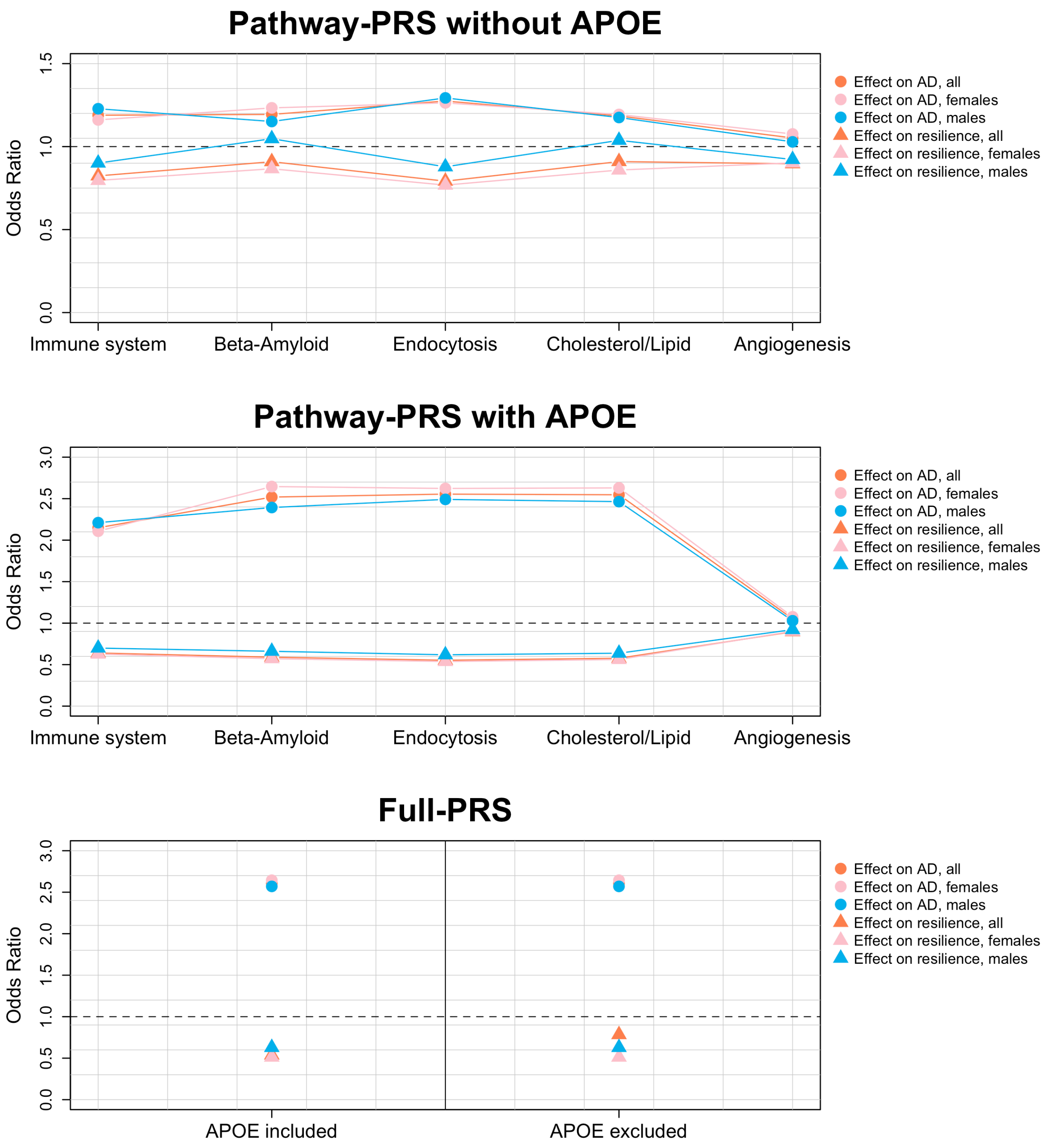

Figure S2: Sex-stratified analysis of the (p)PRS: the figure shows the sex-stratified analyses in the context of the overall analysis, respectively for the pathway-PRS (including and excluding *APOE* variants) and the Full-PRS (including and excluding *APOE* variants). For each PRS (pathway-PRS or Full-PRS) we report both the odds ratio for AD and those for AD-resilience.

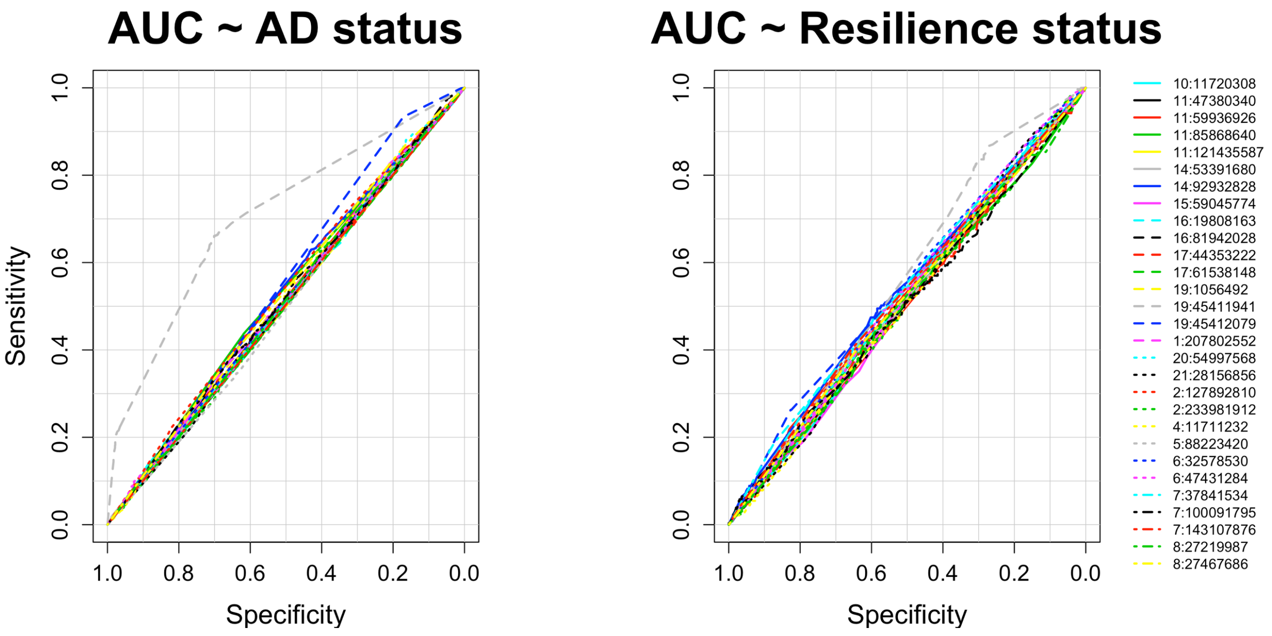

Figure S3: Area under ROC curve for classification of AD or AD-Resilience status, for each variant: the figure shows the quality of the classification of AD and AD-Resilience status using single-variants.

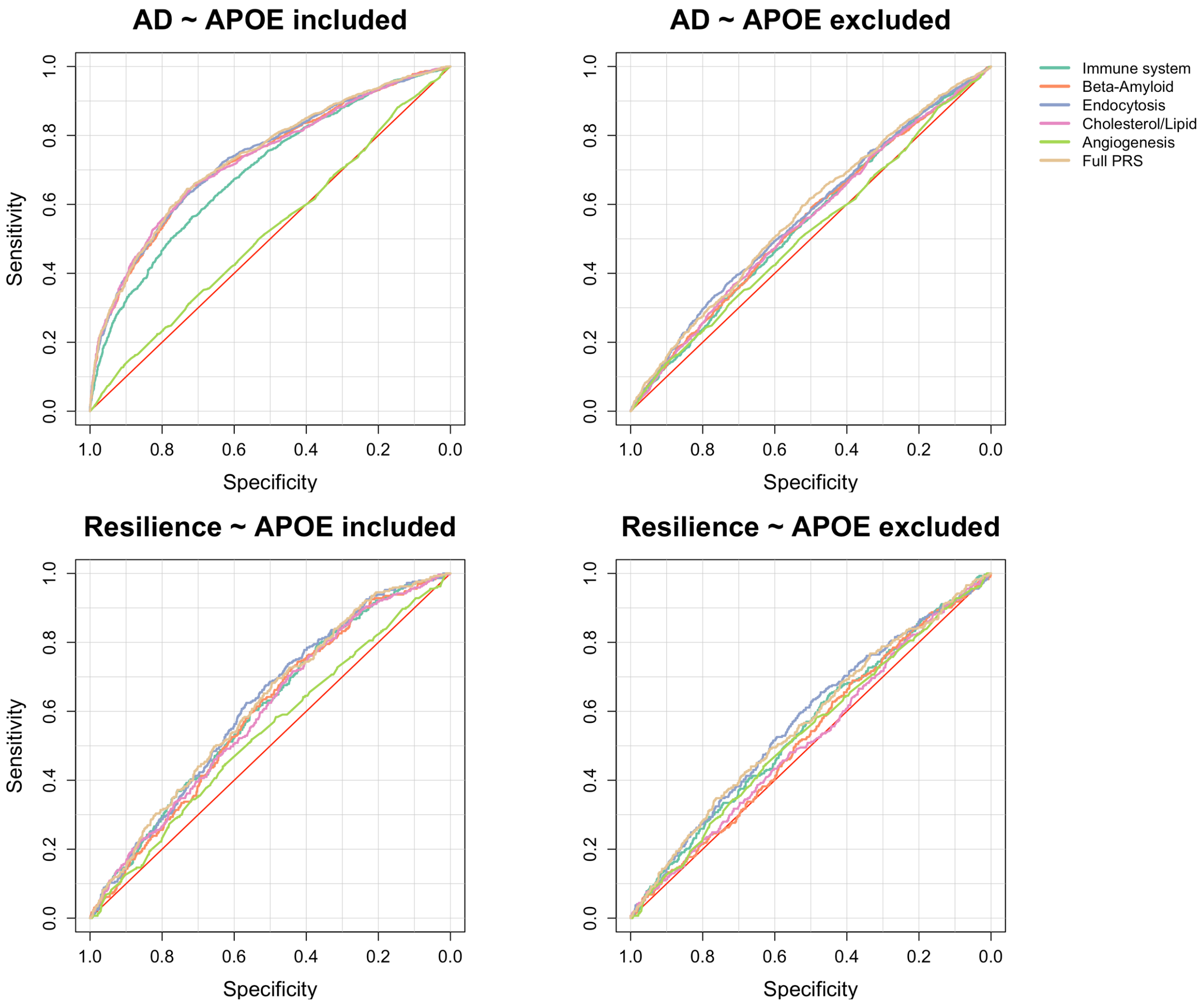

Figure S4: Area under ROC curve for classification of AD and AD-Resilience status, for each PRS and pPRS: the figure shows the quality of the classification of AD and AD-Resilience status using PRS and pPRS.
